# Association of blood culture with carbapenem use in pyogenic liver abscess: a two-center retrospective study

**DOI:** 10.1186/s12873-021-00442-2

**Published:** 2021-05-03

**Authors:** Shuangjun He, Jie Yu, Hairong Wang, Lifeng Wang, Yi Chen, Wei Zhou

**Affiliations:** 1 Department of Emergency, Shanghai Jiao Tong University School of Medicine affiliated Renji Hospital, 2000 Jiangyue Road, Minhang District, Shanghai, 200025 China; 2 Department of Emergency, Shanghai Jiao Tong University School of Medicine affiliated Xinhua Hospital, Shanghai, China

**Keywords:** Pyogenic liver abscess, Blood culture, Carbapenems, Extended-spectrum beta-lactamase, Sepsis

## Abstract

**Background:**

Highly empiric use of carbapenem in pyogenic liver abscess (PLA) is widespread problem. However, few studies have examined the association between blood culture and carbapenem use in patients with PLA in China. Thus, we conducted this observational study.

**Methods:**

The data of patients diagnosed with PLA at two comprehensive tertiary care centers from 2014 to 2020 were retrospectively collected. Demographic and clinical data were analyzed, and univariate and multivariate analyses were performed to investigate the association between blood culture and carbapenem use. Subgroup analysis was conducted to explore whether the effect is different in sepsis.

**Results:**

Blood culture was performed in 110 (46.0%) patients, of whom 44 (40.0%) patients had positive results for bacterial culture. Extended-spectrum beta-lactamase (ESBL)-positive blood culture isolates were detected in 8 (7.3%) patients. The positivity rate of blood culture in sepsis was higher than in non-sepsis (58.1% vs. 32.9%, *P* = 0.015). Fewer patients who had a blood culture received carbapenem treatment in comparison to patients without blood culture (19.1% vs. 31.8%, *P* = 0.026). Multivariate analysis showed that blood culture was independently associated with less carbapenem exposure (adjusted odds ratio [OR] = 0.33, 95% confidence interval [CI]: 0.16–0.68, *P* = 0.003), and this effect remained significant in the sepsis subgroup (adjusted OR = 0.17, 95% CI: 0.05–0.53, *P* = 0.002).

**Conclusion:**

Blood culture had a high positivity rate and was associated with less carbapenem use in PLA, especially those who developed sepsis. More attention should be paid to performing early blood culture and less carbapenem use in PLA.

**Supplementary Information:**

The online version contains supplementary material available at 10.1186/s12873-021-00442-2.

## Background

Pyogenic liver abscess (PLA) is a common infectious disease in the emergency department and its prevalence has increased steadily in China in recent years [1, 2]. With the widespread use of antibiotics and development of new imaging technology and drainage techniques, the mortality rate associated with PLA is slowly declining. PLA is often complicated by sepsis, and according to the current guidelines, adequate antibiotic therapy should be administered as soon as possible, ideally within 1 h [3]. The initial choice of antibiotic is usually based on empirical evidence only as the results of microbiological culture are not readily available. Therefore, emergency clinicians tend to administer wide-spectrum antibiotics. However, unwarranted exposure to antibiotics, especially carbapenems, can inevitably lead to antibiotic resistance, resulting in adverse effects and high costs. Targeted antibiotic therapy for the causative pathogen can effectively reduce these associated complications.

Blood culture can detect pathogenic bacteria in patients with PLA. They are considered the most sensitive method for detecting bacteremia and commonly performed for patients with fever, chills, leukocytosis, focal infections, and sepsis [4]. However, the implementation of blood culture in the emergency department is limited owing to a low positivity rate and the large amount of sample needed (typically 40–50 ml blood). There is also an increasing number of techniques that are currently being used in clinical practice to rapidly test the presence of microorganisms, such as metagenomics next generation sequencing (mNGS), matrix-assisted laser desorption/ionization time of flight (MALDI-TOF), and SeptiFast. Therefore, some researchers have suggested that a blood culture should not be performed for adult patients with isolated fever or leukocytosis without considering the pretest probability [5]. However, some researchers argue that performing a blood culture after administration of antibiotics diminishes their sensitivity and clinical utility [6]. In a previous study, the blood culture positivity in PLA (3–6 cm) was approximately 28% [7]. Therefore, we hypothesized that blood culture could help in scientific and rational medical decision making for antimicrobial management strategies.

Thus far, limited studies have evaluated the relationship between performing blood culture and carbapenem use in PLA. Therefore, this study aimed to characterize blood culture in PLA and explore its association with carbapenem use. We investigated whether the empiric use of carbapenem for PLA is the best option in the emergency room setting or perhaps routine blood culture with extended-spectrum beta-lactamase (ESBL) is a better option.

## Methods

### Study design and patients

**Fig. 1:**
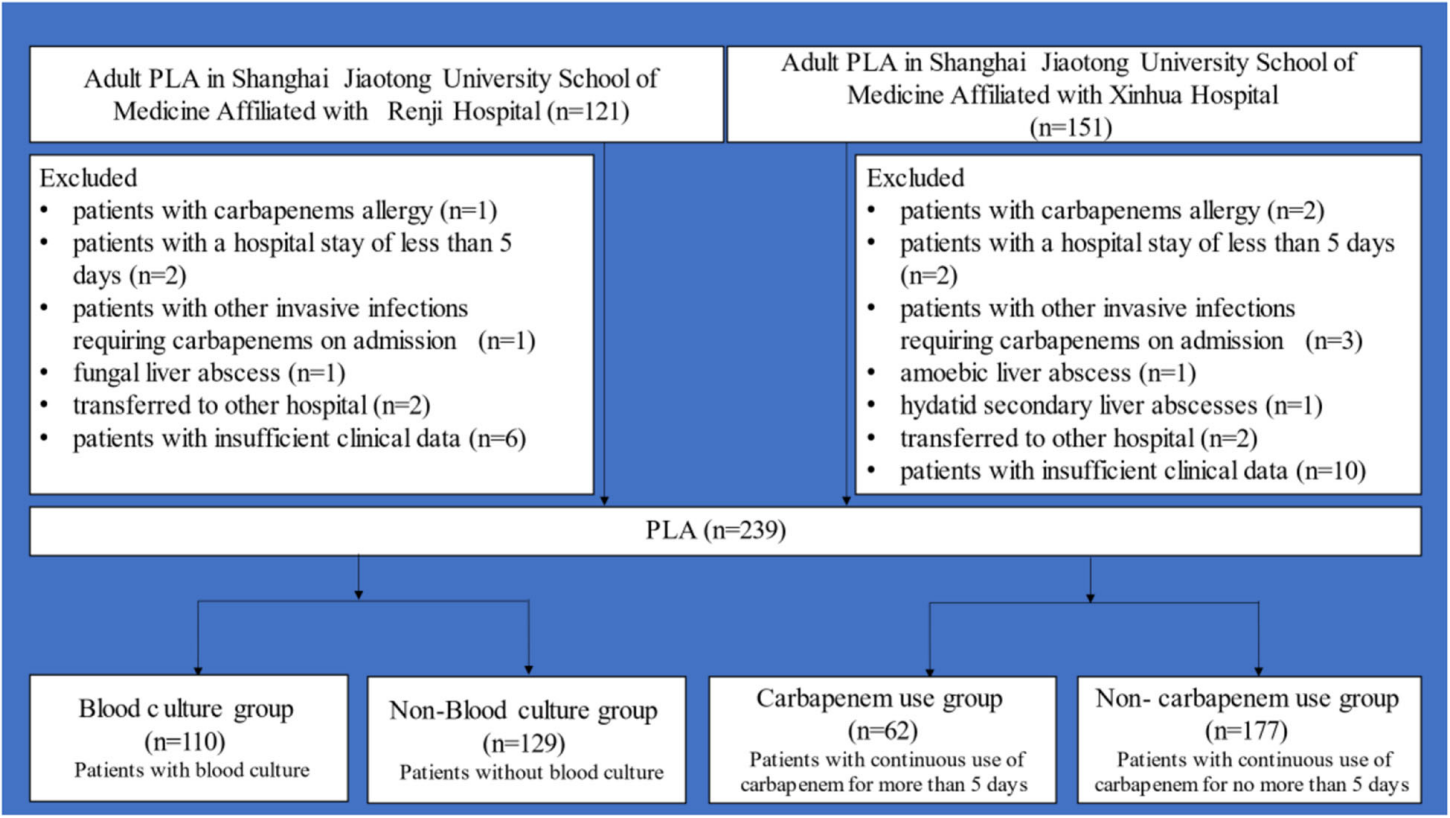
Flow chart of the patients with PLA reviewed in the study during Jan 2014 to Mar 2020

This retrospective cohort study was conducted at two comprehensive tertiary care centers in Shanghai that had 604 and 2090 beds. All adult patients with PLA who visited the emergency department from January 2014 to March 2020 were reviewed. Exclusion criteria were as follows: a) carbapenem allergy; b) hospital stay < 5 days; c) complications with other invasive infections requiring carbapenem administration on admission; d) amoebic liver abscess, fungal liver abscess, and hydatid secondary liver abscess; e) transferred from another hospital; and f) insufficient clinical data (Fig. 1). This study was approved by the ethics committee of both hospitals. Owing to the retrospective nature of the study, the requirement for informed consent was waived.

### Data collection and variable definitions

The medical records of all eligible patients discharged from the two centers with a diagnosis of PLA, defined by the International Classification of Disease, 10th Revision code K75.0, were reviewed.

Anonymized patient data were collected by two trained observers using a prespecified case report form. Data on demographic characteristics, coexisting disease, clinical presentations, laboratory findings, radiological findings, microbiologic data, antibiotics data, complications, and clinical outcomes were reviewed.

Hepatobiliary benign diseases included fatty liver disease, hepatitis, liver cirrhosis, biliary stone diseases, and biliary tract inflammation. Hepatobiliary malignant diseases consisted of hepatocellular carcinoma and cholangiocarcinoma. Abdominal surgery history included cholecystectomy, biliary tract surgery, gastrointestinal surgery, hepatectomy, gynecologic surgery, and pancreatic surgery. Clinical presentations included symptoms at admission and changes during hospitalization, such as daily body temperature. Laboratory findings included hematologic and biochemical findings. Radiological findings, including abscess size, number, and location, were assessed using abdominal ultrasonography, computed tomography, or magnetic resonance imaging. Microbiological data, such as time of blood culture, time of the oral preliminary report, time of blood culture formal report, microbial strain, and extended spectrum beta-lactamases (ESBL) positivity, were collected. Data on antibiotics use included antibiotic type (such as cephalosporins, piperacillin/tazobactam, metronidazole, quinolones, glycopeptides, and carbapenems), dose, course, reasons for antibiotic changes, and adjusted regimens. Carbapenem use was defined as continuous exposure to carbapenems for more than 5 days. Since microbial sensitivity testing results were usually obtained within 6 days (> 95% blood culture and drug sensitivity results were officially reported on day 6 of ordering the culture), the antimicrobial regimen was subsequently maintained or adapted according to the results. The severity of illness on admission was evaluated with the Sequential Organ Failure Assessment (SOFA) scoring system in the first 24-h on admission. Complications included sepsis and sepsis shock based on the Sepsis-3 definition [8]. Clinical outcomes were as follows: a) in-hospital mortality, defined as PLA-related mortality in hospital; b) length of hospitalization, defined as the number of days of hospital stay; and c) clinical recovery, defined as the number of days until the body temperature returned to < 37.3 °C or symptomatic improvement after discharge.

### Blood culture

Aerobic and anaerobic cultures were performed for the blood samples. Species identification and antimicrobial susceptibility were tested using VITEK automated systems (bioMérieux Vitek, Hazelwood, MO, USA) and interpreted according to the Clinical and Laboratory Standards Institute (CLSI) criteria.

Blood culture results were classified as positive or negative based on the presence or absence of bacterial growth, respectively. The provisional oral report was provided to clinicians immediately after obtaining strain staining results (usually the next day). Blood culture positivity was defined as the detection of a microbiological pathogen within 6 days of incubation. The results of blood culture showing presence bacterial growth were considered negative if the microbiological pathogen could not be identified. Each positive blood culture result was assessed for contamination (false positivity) according to the established criteria [9]. Thereafter, all false-positive culture results were coded as negative.

Phenotypical confirmation of ESBL detection and susceptibility to ESBL-positive isolates were evaluated using the disk diffusion technique in our clinical microbiology laboratories, as recommended by the CLSI.

### Statistical analysis

Data are presented as number (%) for categorical variables and as mean and standard deviation or median (interquartile range) for continuous variables with normal distribution and non-normal distribution, respectively. The independent Student’s t-test or nonparametric Mann-Whitney test was used to analyze continuous variables. The Pearson’s chi-square test or Fisher’s exact test was used for categorical variables. Normality was tested using the Shapiro-Wilk test.

Univariate and multivariate analyses were performed to identify significant factors related to carbapenem use in patients with PLA. Subgroup analyses were performed according to whether the patients also developed sepsis. Clinically relevant variables and those with *P*-values < 0.10 in the univariate analyses were included in the multivariable logistic regression model [10, 11]. The odds ratios (ORs) and 95% confidence intervals (95% CIs) were estimated using the logistic regression model. *P* < 0.05 was considered statistically significant. Statistical analyses were performed using IBM SPSS version 22.0 (SPSS Inc., Chicago, IL, USA).

## Results

### Demographic and clinical characteristics

In total, 272 patients with PLA were admitted to the two hospitals from January 2014 to March 2020. Of these, 239 patients met the inclusion criteria (109 and 130 from Renji and Xinhua hospitals, respectively), with 110 patients (46.0%) in the blood culture group and 129 (54.0%) in the non-blood culture group. The mean age of the blood culture group was 63.1 years, and 56.4% were male. The number of coexisting diseases (diabetes mellitus, hepatobiliary benign disease, and abdominal surgery history) was significantly higher in the blood culture group than in the non-blood culture group. Fever was the most frequent symptom (90.4%), followed by abdominal pain (31.0%) in all the patients. There were no significant differences in symptoms at presentation, abscess characteristics, SOFA scores, sepsis, or septic shock between the groups. However, the group with blood culture had lower C-reactive protein (CRP) levels than the non-blood culture group (107.2 ± 63.8 vs. 130.3 ± 59.7 mg/L, *P* = 0.004).

Carbapenems were less frequently used in the blood culture group than in the non-blood culture group (21 [19.1%] vs. 41 [31.8%] patients, *P* = 0.026). In total, 182 patients (76.2%) received combination therapy with antibiotics. The other antibiotic regimes and the proportion of combination antibiotic therapy were similar between groups. The length of hospitalization was shorter in the blood culture group than in the non-blood culture group (15.0 [inter-quartile range (IQR): 13.0–22.0) vs. 13.0 [IQR: 9.0–19.0] days, *P* < 0.001). Most patients (96.2%) recovered, and only nine of the 239 patients (3.8%) died during the hospital stay. Four patients died of severe general sepsis or septic shock, three died of cachexia and subsequent multiple organ failure, one died of inadequate antibiotic coverage, and one died of severe hypertonic hyperglycemic syndrome. However, in-hospital mortality between the two groups did not differ (Table 1).

**Table 1:** Baseline characteristics

Variables	Blood Culture (*N* = 110)	Non-Blood Culture (*N* = 129)	*P* value	Carbapenem use (*N* = 62)	Non-Carbapenem use (*N* = 177)	*P* value
**Age**, mean ± SD (years)	63.1 ± 13.4	64.3 ± 13.3	0.492	67.0 ± 12.9	62.6 ± 13.4	0.026
**Sex (male)**, n (%)	62 (56.4%)	73 (56.6%)	0.972	33 (53.2%)	102 (57.6%)	0.547
**Coexisting diseases**, n (%)						
Diabetes mellitus	68 (61.8%)	62 (48.1%)	0.033	31 (50.0%)	94 (53.1%)	0.673
Hepatobiliary benign disease	49 (44.6%)	39 (30.2%)	0.022	18 (29.0%)	70 (39.6%)	0.140
Underlying malignancy	10 (9.1%)	9 (7.0%)	0.547	5 (8.1%)	14 (7.9%)	0.969
Abdominal surgery history	27 (24.6%)	16 (12.4%)	0.015	7 (11.3%)	36 (20.3%)	0.110
**Symptoms on admission**, n (%)						
Fever (BT > 37.3 °C)	98 (89.1%)	118 (91.5%)	0.534	57 (91.9%)	159 (89.8%)	0.629
Abdominal pain	37 (33.6%)	37 (28.7%)	0.409	21 (33.9%)	53 (29.9%)	0.565
General Weakness	21 (19.1%)	25 (19.4%)	0.955	16 (25.8%)	30 (17.0%)	0.128
**Abscess location**, n (%)			0.449			0.692
Right lobe	87 (79.1%)	93 (72.1%)		45 (72.6%)	135 (76.3%)	
Left lobe	19 (17.3%)	29 (22.5%)		13 (21.0%)	35 (19.8%)	
Both lobes	4 (3.6%)	7 (5.4%)		4 (6.5%)	7 (4.0%)	
**Abscess number**, n (%)			0.054			0.146
Solitary abscess	99 (90.8%)	106 (82.2%)		50 (80.7%)	155 (88.1%)	
Multiple abscess	10 (9.2%)	23 (17.8%)		12 (19.4%)	21 (11.9%)	
**Average size of abscess** (cm)	6.1 ± 2.8	5.9 ± 2.4	0.515	5.9 ± 2.8	6.1 ± 2.6	0.632
**Laboratory findings**						
Leucocytes (×10^9^/L)	10.3 ± 4.3	11.4 ± 5.1	0.063	12.5 ± 4.8	10.4 ± 4.7	0.002
C-reactive protein (mg/L)	107.2 ± 63.8	130.3 ± 59.7	0.004	130.9 ± 61.9	115.8 ± 62.5	0.102
Procalcitonin (ng/ml)	5.0 (0.4–9.8)	4.3 (0.5–11.6)	0.759	6.1 (1.5–12.7)	3.3 (0.3–10.5)	0.065
**Time of oral preliminary blood culture report** (days)	1.0 ± 0.2	NA	NA	NA	NA	NA
**Time of blood culture result** (days)	6.0 ± 0.5	NA	NA	NA	NA	NA
**Positive Blood Culture rate**, n (%)	44 (40.0%)	NA	NA	NA	NA	NA
**Positive ESBL rate**, n (%)	8 (7.3%)	NA	NA	NA	NA	NA
**SOFA scores**	0.0 (0.0–2.0)	0.0 (0.0–2.0)	0.589	1.0 (0.0–2.0)	0.0 (0.0–1.0)	0.002
**Sepsis**, n (%)	31 (28.2%)	36 (27.9%)	0.962	28 (45.2%)	39 (22.0%)	< 0.001
**Septic shock**, n (%)	2 (1.8%)	7 (5.4%)	0.144	7 (11.3%)	2 (1.1%)	< 0.001
**Antibiotics treatment**, n (%)						
Cephalosporins	99 (90.0%)	118 (91.5%)	0.695	48 (77.4%)	169 (95.5%)	< 0.001
Piperacillin/tazobactam	4 (3.6%)	7 (5.4%)	0.510	2 (3.2%)	9 (5.1%)	0.733
Quinolones	39 (35.5%)	45 (34.9%)	0.927	12 (19.4%)	72 (40.7%)	0.002
Metronidazole	30 (27.3%)	48 (37.2%)	0.102	9 (14.5%)	69 (39.0%)	< 0.001
Glycopeptides	4 (3.6%)	4 (3.1%)	0.819	2 (3.2%)	6 (3.4%)	0.951
Carbapenem	21 (19.1%)	41 (31.8%)	0.026	62 (100.0%)	NA	NA
**Antibiotics option**, n (%)			0.325			0.002
Combined	87 (79.1%)	95 (73.7%)		54 (87.1%)	118 (66.7%)	
Single	23 (20.9%)	34 (26.4%)		8 (12.9%)	59 (33.3%)	
**In-hospital mortality**, n (%)	4 (3.6%)	5 (3.9%)	0.923	6 (9.7%)	3 (1.7%)	0.004
**Length of hospitalization** (days)	13.0 (9.0–19.0)	15.0 (13.0–22.0)	< 0.001	19.5 (14.0–25.8)	14.0 (10.0–19.0)	< 0.001
Clinical recovery (days)	2.0 (1.0–4.0)	2.0 (1.0–4.0)	0.794	3.0 (2.0–6.0)	2.0 (1.0–4.0)	0.075

*BT* body temperature; *ESBL* extended-spectrum beta-lactamase; *NA* not applicable; *SOFA* Sequential Organ Failure Assessment;

In addition, the mean age of the carbapenem use group was significantly higher than the non-carbapenem use group (67.0 years vs. 62.6 years, *P* = 0.026). There were no differences between comorbidity, symptoms on admission, abscess characteristics, or laboratory data of both groups expect leucocytes. However, the carbapenem use group had higher leucocytes, SOFA scores, proportion of sepsis, septic shock, mortality, and longer hospital stay than the non-carbapenem use group (Table 1). After adjusting for potential confounding factors in the multivariate models, carbapenem use was related to longer hospital stay (*P* = 0.0128) rather than increased mortality (*P* = 0.2005) (Supplementary appendix Table S1).

### Microbiological characteristics

Among the 110 patients in the blood culture group, 44 (40.0%) patients had a positive bacterial culture result and 8 (7.3%) patients were ESBL positive. The mean time for obtaining the oral preliminary blood culture report and formal report was 1 day and 6 days, respectively (Table 1). *Klebsiella pneumonia* (32/44 patients, 72.7%) was the most common pathogen, followed by *Streptococcus* spp. (4/44 patients, 9.1%). There were 4 (9.1%) patients with polymicrobial infection and 40 (90.9%) patients with monomicrobial infection. Furthermore, the blood culture positivity rate was significantly higher in the sepsis group than in the non-sepsis group (58.1% vs. 32.9%, *P* = 0.015) (Table 2).

**Table 2:** Microbiological characteristics in blood culture group

Organism cultured, n (%)	Blood culture (***N*** = 110)		***P*** value
	Sepsis (***N*** = 31)	Non-sepsis (***N*** = 79)	
**Positive**	18 (58.1%)	26 (32.9%)	0.015
*Klebsiella pneumonia*	12 (66.7%) ^a^	20 (76.9%) ^a^	0.453
*Streptococcus* spp.	3 (16.7%) ^a^	1 (3.9%)	0.146
*Staphylococcus* spp.	1 (5.6%) ^a^	1 (3.9%)	0.789
*Escherichia coli*	1 (5.6%)	2 (7.7%)	0.782
*Enterococcus* spp.	1 (5.6%)	1 (3.9%) ^a^	0.789
*Enterobacter* spp.	1 (5.6%)	2 (7.7%) ^a^	0.782
*Pseudomonas aeruginosa*	1 (5.6%) ^a^	1 (3.9%)	0.789
**ESBLs positive**	4 (12.9%)	4 (5.1%)	0.154
**Monomicrobial infection**	16 (88.9%)	24 (92.3%)	0.698
**Polymicrobial infection**	2 (11.1%)	2 (7.7%)	0.698
**Negative**	13 (41.9%)	53 (67.1%)	0.015

^a^There were four cases of polymicrobial infection in the blood culture group

### Association between blood culture and carbapenem use

Performing a blood culture was associated with a lower exposure to carbapenems (OR = 0.50, 95% CI: 0.28–0.93, *P* = 0.026). Sepsis, high leucocyte count, and elderly age were significantly associated with more carbapenem use in univariate analysis. The multivariate analysis showed that blood culture was associated with less carbapenem use, although the relationship was borderline significant (OR = 0.53, 95% CI: 0.28–1.00, *P* = 0.050) (Supplementary appendix Table S2).

After adjusting for potential confounding factors in the multivariate models, the association of blood culture with carbapenem use remained significant (adjusted OR = 0.33, 95% CI: 0.16–0.68, *P* = 0.003) (Table 3). Finally, we performed subgroup analyses according to whether the patient developed sepsis. In patients with sepsis, the adjusted OR was 0.17 (95% CI: 0.05–0.53, *P* = 0.002). However, in non-sepsis patients, the adjusted OR was 0.65 (95% CI: 0.22–1.97, *P* = 0.451), which did not reach statistical significance.

**Table 3:** Multivariate models for carbapenem use and blood culture stratified by sepsis

Group	Carbapenem use	Model 1^**a**^		Model 2^**b**^		Model 3^**c**^	
		Odds ratio (95% CI)	P value	Odds ratio (95% CI)	P value	Odds ratio (95% CI)	P value
**All population**	62 (25.9%)	0.50 (0.28–0.93)	0.026	0.49 (0.26–0.91)	0.025	0.33 (0.16–0.68)	0.003
**Sepsis**	28 (41.8%)	0.29 (0.12–0.69)	0.005	0.29 (0.12–0.68)	0.005	0.17 (0.05–0.53)	0.002
**Non-sepsis**	34 (19.8%)	1.01 (0.38–2.68)	0.982	1.03 (0.38–2.80)	0.952	0.65 (0.22–1.97)	0.451

^a^: Unadjusted

^b^: Adjusted for age and gender

^c^: Adjusted for age, gender, leucocyte count, and C-reaction protein level, ESBL, biliary disease, gastrointestinal malignancy

*CI* confidence intervals;

## Discussion

This retrospective cohort study analyzed data on patients with PLA over 6 years from databases from two centers and found that performing blood culture was associated with a lower rate of carbapenem exposure in patients with PLA, especially in those complicated with sepsis. The higher etiology positivity rate and the faster antibiotic degradation based on drug sensitivity results might play a role in explaining this correlation. To the best of our knowledge, this is the first study to evaluate the relationship between performing blood culture and carbapenem use in patients with PLA. While previous studies have reported the benefits of blood culture in many infectious diseases, these results are slightly contradictory with the routine submission of blood culture [6, 12, 13]. In this study, we have used the data from two emergency centers to help clinicians make decisions regarding the utility of blood culture in the early stages of infection, while keeping in line with the current sepsis guidelines [4, 8]. Altogether, the present data support carbapenem-sparing options.

The ESBL positivity rate of 8/110 (7.3%) observed in our study was lower than that reported in former studies [14]. We found that most patients in this study had more underlying diseases, recurrent episodes of infection, and broad-spectrum antibiotics exposure. PLA caused by ESBL producing organisms from community-acquired infection is rare in China, which is consistent with the study by Lin et al. Lin et al. also identified three specific genome regions in PLA strains; all PLA strains and non-invasive strains were ampicillin-resistant and cefotaxime susceptible, and none were ESBL producing. The study also suggested that ESBLs are not associated with PLA [15]. Furthermore, in our study, the mortality rate in patients without microbiological evidence of infection was similar to that in patients with microbiological evidence of infection, which is in line with previous studies [16, 17]. Therefore, we believe that it is not necessary to prescribe carbapenems for PLA patients with or without sepsis.

Blood culture plays a critical role in sepsis management and should be recommended for patients with sepsis [4]. Obtaining a blood culture during antibiotic therapy is associated with a significantly low pathogen detection rate. To maximize utility, at least two sets of blood culture should be obtained before initiating antibiotic therapy [18], which is consistent with the recent data reported by Dellinger et al. [19]. There was a considerable proportion of patients with PLA who did not undergo the blood culture test in the current study. Possible reasons include the unwillingness of patients to cooperate with the examination, neglect of etiology examination due to initial effective treatment, and the application of new techniques such as mNGS and MALDI-TOF.

In our study, the general baseline characteristics were almost similar in the blood culture and non-blood culture groups; however, clinicians tended to order blood culture in PLA patients with diabetes mellitus, hepatobiliary benign diseases, or abdominal surgery history. These differences could be attributed to PLA patients with coexisting diseases becoming more common in hospitals, particularly since previous researchers have demonstrated that diabetes mellitus results in an increased risk of PLA [20, 21]. Li et al. performed blood culture for 118 patients with PLA and found that patients with diabetes mellitus were significantly more likely to have a positive blood culture result [21]. Furthermore, there was a significant difference in the CRP levels, possibly indicating that the severity of infection was higher in the blood culture group than in the non-blood culture group, suggesting that clinicians tended to identify pathogens in cases where infection was considered severe. We also illustrated that carbapenem use was related to elderly age, worse severity of illness and longer hospitalization. This might be because clinicians usually prescribe carbapenems in more severe cases for wider coverage and stronger elimination of bacteria. It might suggest alternative antibiotic policy and carbapenem-sparing options and call for reduction of carbapenems use as much as possible.

The global spread of ESBLs has led to a significant increase in carbapenem use. Moreover, carbapenems are usually used as front-line treatment for gram-negative bacterial infections because of its progressive resistance among other ESBLs. Clinicians tend to start carbapenems in patients with severe cases, unsatisfactory initial treatment, or a history of drug resistance in order to cover a broader spectrum of pathogens. However, as documented in large surveillance studies, carbapenem resistance has been increasing [22]. Furthermore, there are significant variations in the usage of antibiotics and the ordering of blood culture between hospitals and clinicians. Our study has contributed to the understanding of the relationship between performing blood culture and carbapenem use. Multiple regression analysis was used to adjust many confounding factors and stratify patients with or without sepsis. We found that carbapenem use was reduced by 83% in patients who underwent a blood culture test than those who did not undergo a blood culture in sepsis, which might help improve drug resistance and rational prescription of antibiotics.

Our study has some limitations that must be taken into consideration. First, the study was retrospective in nature, and procedures related to performing blood culture with carbapenem administration were not standardized, which reflects the current real-world practice, especially in many developing countries, such as China. Second, there has been no scientific consensus about carbapenem exposure, and our results might have been affected by the culture time. Third, despite careful adjustment for several confounding factors, unmeasured confounders could have biased our results and the subsequent conclusions. Finally, the present study may only reflect a local problem of antibiotic policies. Strong conclusions cannot be made because the sample size was small and the results reported in two emergency medical centers in Shanghai cannot be extrapolated to other populations with different epidemiological or clinical settings.

## Conclusion

In conclusion, blood culture is a useful method for detecting pathogenic bacteria and may reduce inappropriate carbapenem use, particularly in PLA complicated with sepsis. Thus, we call for more blood culture, more blood culture before antibiotics and less carbapenem use in PLA therapy in emergency room setting.

## Supplementary Information

**Additional file 1.**

## Data Availability

The datasets used and/or analyzed during the current study are available from the corresponding author on reasonable request.

## Abbreviations

PLA: Pyogenic liver abscess
ESBL: Extended-spectrum beta-lactamase
OR: Odds ratio
CI: Confidence interval
mNGS: Metagenomics next generation sequencing
MALDI-TOF: Matrix-assisted laser desorption/ionization time of flight
SOFA: Sequential Organ Failure Assessment
CLSI: Clinical and Laboratory Standards Institute
CRP: C-Reactive protein
IQR: Inter-quartile range

## References

[1] Meddings L, Myers RP, Hubbard J, Shaheen AA, Laupland KB, Dixon E, Coffin C, Kaplan GG (2010). A population-based study of pyogenic liver abscesses in the United States: incidence, mortality, and temporal trends. Am J Gastroenterol.

[2] Sharma A, Mukewar S, Mara KC, Dierkhising RA, Kamath PS, Cummins N (2018). Epidemiologic factors, clinical presentation, causes, and outcomes of liver abscess: a 35-year Olmsted County study. Mayo Clin Proc Innov Qual Outcomes.

[3] Rhodes A, Evans LE, Alhazzani W, Levy MM, Antonelli M, Ferrer R, Kumar A, Sevransky JE, Sprung CL, Nunnally ME, Rochwerg B, Rubenfeld GD, Angus DC, Annane D, Beale RJ, Bellinghan GJ, Bernard GR, Chiche JD, Coopersmith C, de Backer DP, French CJ, Fujishima S, Gerlach H, Hidalgo JL, Hollenberg SM, Jones AE, Karnad DR, Kleinpell RM, Koh Y, Lisboa TC, Machado FR, Marini JJ, Marshall JC, Mazuski JE, McIntyre LA, McLean AS, Mehta S, Moreno RP, Myburgh J, Navalesi P, Nishida O, Osborn TM, Perner A, Plunkett CM, Ranieri M, Schorr CA, Seckel MA, Seymour CW, Shieh L, Shukri KA, Simpson SQ, Singer M, Thompson BT, Townsend SR, van der Poll T, Vincent JL, Wiersinga WJ, Zimmerman JL, Dellinger RP (2017). Surviving Sepsis campaign: international guidelines for Management of Sepsis and Septic Shock: 2016. Crit Care Med.

[4] Long B, Koyfman A (2016). Best clinical practice: blood culture utility in the emergency department. J Emerg Med.

[5] Coburn B, Morris AM, Tomlinson G, Detsky AS (2012). Does this adult patient with suspected bacteremia require blood cultures. JAMA.

[6] Lee A, Mirrett S, Reller LB, Weinstein MP (2007). Detection of bloodstream infections in adults: how many blood cultures are needed. J Clin Microbiol.

[7] He S, Yu J, Wang H, Chen X, He Z, Chen Y (2020). Percutaneous fine-needle aspiration for pyogenic liver abscess (3-6 cm): a two-center retrospective study. BMC Infect Dis.

[8] Seymour CW, Liu VX, Iwashyna TJ, Brunkhorst FM, Rea TD, Scherag A, Rubenfeld G, Kahn JM, Shankar-Hari M, Singer M, Deutschman CS, Escobar GJ, Angus DC (2016). Assessment of clinical criteria for Sepsis: for the third international consensus definitions for Sepsis and septic shock (Sepsis-3). JAMA.

[9] Hall KK, Lyman JA (2006). Updated review of blood culture contamination. Clin Microbiol Rev.

[10] Ren Y, Wang H, Chang Z, Liu Z (2020). Clinical and computed tomography features of extended-spectrum β-lactamase-producing Klebsiella pneumoniae liver abscess. BMC Infect Dis.

[11] Shi SH, Zhai ZL, Zheng SS (2018). Pyogenic liver abscess of biliary origin: the existing problems and their strategies. Semin Liver Dis.

[12] Buehler SS, Madison B, Snyder SR, Derzon JH, Cornish NE, Saubolle MA, Weissfeld AS, Weinstein MP, Liebow EB, Wolk DM (2016). Effectiveness of practices to increase timeliness of providing targeted therapy for inpatients with bloodstream infections: a laboratory medicine best practices systematic review and meta-analysis. Clin Microbiol Rev.

[13] Cargill J, Etherington C, Peckham D, Conway S, Denton M (2012). Bloodstream infections in cystic fibrosis: nine years of experience in both adults and children. J Cyst Fibros.

[14] Lo JZ, Leow JJ, Ng PL, Lee HQ, Mohd Noor NA, Low JK (2015). Predictors of therapy failure in a series of 741 adult pyogenic liver abscesses. J Hepatobiliary Pancreat Sci.

[15] Lin TL, Tang SI, Fang CT, Hsueh PR, Chang SC, Wang JT (2006). Extended-spectrum beta-lactamase genes of Klebsiella pneumoniae strains in Taiwan: recharacterization of shv-27, shv-41, and tem-116. Microb Drug Resist.

[16] Phua J, Ngerng W, See K, Tay C, Kiong T, Lim H, Chew M, Yip H, Tan A, Khalizah H, Capistrano R, Lee K, Mukhopadhyay A (2013). Characteristics and outcomes of culture-negative versus culture-positive severe sepsis. Crit Care.

[17] Vincent JL, Sakr Y, Sprung CL, Ranieri VM, Reinhart K, Gerlach H, Moreno R, Carlet J, le Gall JR, Payen D, Sepsis Occurrence in Acutely Ill Patients Investigators (2006). Sepsis in European intensive care units: results of the SOAP study. Crit Care Med.

[18] Scheer CS, Fuchs C, Gründling M, Vollmer M, Bast J, Bohnert JA, Zimmermann K, Hahnenkamp K, Rehberg S, Kuhn SO (2019). Impact of antibiotic administration on blood culture positivity at the beginning of sepsis: a prospective clinical cohort study. Clin Microbiol Infect.

[19] Dellinger RP, Levy MM, Rhodes A, Annane D, Gerlach H, Opal SM, Sevransky JE, Sprung CL, Douglas IS, Jaeschke R, Osborn TM, Nunnally ME, Townsend SR, Reinhart K, Kleinpell RM, Angus DC, Deutschman CS, Machado FR, Rubenfeld GD, Webb SA, Beale RJ, Vincent JL, Moreno R, Surviving Sepsis Campaign Guidelines Committee including the Pediatric Subgroup (2013). Surviving sepsis campaign: international guidelines for management of severe sepsis and septic shock: 2012. Crit Care Med.

[20] Thomsen RW, Jepsen P, Sørensen HT (2007). Diabetes mellitus and pyogenic liver abscess: risk and prognosis. Clin Infect Dis.

[21] Li W, Chen H, Wu S, Peng J (2018). A comparison of pyogenic liver abscess in patients with or without diabetes: a retrospective study of 246 cases. BMC Gastroenterol.

[22] Esterly JS, Wagner J, McLaughlin MM, Postelnick MJ, Qi C, Scheetz MH (2012). Evaluation of clinical outcomes in patients with bloodstream infections due to gram-negative bacteria according to carbapenem MIC stratification. Antimicrob Agents Chemother.

