# Supplementary material for: Association of blood culture with carbapenem use in pyogenic liver abscess: a two-center retrospective study
Source: BMC Emerg Med. 2021 May 3;21:58. doi: 10.1186/s12873-021-00442-2 (PMC8091740; doi:10.1186/s12873-021-00442-2)
Supplement: Supplementary file 1 — Additional file 1. [file 12873_2021_442_MOESM1_ESM.docx]

**Supplementary appendix**

Table S1. Multivariate models for relationship between outcomes and carbapenem use

|  | **Model 1^a^** | | **Model 2^b^** | | **Model 3^c^** | |
| --- | --- | --- | --- | --- | --- | --- |
|  | **Odds ratio (95% CI)** | **P value** | **Odds ratio (95% CI)** | **P value** | **Odds ratio (95% CI)** | **P value** |
| Mortality | 6.21 (1.50-25.66) | 0.0116 | 4.89 (1.09-22.00) | 0.0387 | 3.90 (0.49-31.25) | 0.2005 |
| LOS | 5.71 (2.51, 8.91) | 0.0006 | 5.51 (2.28, 8.74) | 0.0010 | 4.58 (1.00, 8.16) | 0.0128 |

a: Unadjusted

b: Adjusted for age and gender

c: Adjusted for age, gender, leucocyte count, and C-reaction protein level, ESBL, sepsis, blood culture and underlying malignancy

CI: confidence intervals;

LOS: length of hospitalization

Table S2. Univariate and multivariate analysis of carbapenem use

| **Variables** | **Univariate analysis** | | | **Multivariate analysis** | | |
| --- | --- | --- | --- | --- | --- | --- |
|  | **Odds ratio** | **95% CI** | **P value** | **Odds ratio** | **95% CI** | **P value** |
| **Age** (per 10 years) | 1.03 | 1.00-1.05 | 0.027 | 1.22 | 0.96-1.55 | 0.114 |
| **Gender** (male) | 1.20 | 0.60-2.14 | 0.548 |  |  |  |
| **Diabetes mellitus** | 0.88 | 0.49-1.58 | 0.673 |  |  |  |
| **Hepatobiliary benign disease** | 0.60 | 0.30-1.20 | 0.141 |  |  |  |
| **Underlying** **malignancy** | 1.02 | 0.35-2.96 | 0.969 |  |  |  |
| **Abdominal surgery history** | 0.50 | 0.21-1.19 | 0.116 |  |  |  |
| **Abscess size** (≥5cm) | 0.97 | 0.87-1.09 | 0.631 |  |  |  |
| **Abscess location** (right lobe) | 0.82 | 0.43-1.59 | 0.692 |  |  |  |
| **Abscess number** (solitary) | 1.77 | 0.81-3.86 | 0.146 |  |  |  |
| **Blood culture** | 0.50 | 0.28-0.93 | 0.026 | 0.53 | 0.28-1.00 | 0.050 |
| **Pus culture** | 0.77 | 0.43-1.38 | 0.384 |  |  |  |
| **Leucocytes** (per 1×10^9^/L) | 1.09 | 1.03-1.16 | 0.004 | 1.07 | 1.01-1.14 | 0.0267 |
| **C-reactive protein** (≥100mg/L) | 1.00 | 1.00-1.01 | 0.103 |  |  |  |
| **Procalcitonin** (≥0.5ng/ml) | 1.01 | 0.99-1.04 | 0.416 |  |  |  |
| **Sepsis** | 2.91 | 1.58-5.38 | <0.001 | 2.67 | 1.41-5.06 | 0.003 |

CI: confidence intervals; ESBL: extended-spectrum beta-lactamase; NA: not applicable;
